# Prevalence of intestinal parasitic infections in children under the age of 5 years attending the Debre Birhan referral hospital, North Shoa, Ethiopia

**DOI:** 10.1186/s13104-018-3166-3

**Published:** 2018-01-22

**Authors:** Telanesh Zemene, Melashu Balew Shiferaw

**Affiliations:** Parasitology Reference Laboratory, Amhara Public Health Institute, P.O. Box 641, Bahir Dar, Ethiopia

**Keywords:** Parasite, Protozoa, Helminth, Under-five children, Debre Birhan

## Abstract

**Objective:**

Intestinal parasitic infection is one of the major childhood health problems in developing countries. In Ethiopia, epidemiological data for several localities is limited. Hence, the aim of this study is to assess intestinal parasitic infections among under-five children attending in Debre Birhan referral hospital, which could help to decrease morbidity and mortality in children. A cross-sectional study was conducted in February, 2014. Stool specimens were collected and examined using concentration method.

**Results:**

Out of the 247 under-five children participated, 17.4% (95% CI 12.7–22.1%) of the children were infected with at least one or more protozoa parasites (14.2% [95% CI 9.9–18.5%]) and helminthes (3.2% [95% CI 1.0–5.4%]). *Giardia lamblia* (8.5%), *Entamoeba histolytica/dispar* (5.7%), *Trichuris trichiura* (1.6%) and *Ascaris lumbricoides* (1.2%) were the most identified parasites. Parasitic infection was higher in children who had source of drinking water from the river (36.8%), among children from mothers with poor hand washing practice (31.7%), and among children born from illiterate mothers (27.5%). This revealed that intestinal parasites affect the health of under-five children in the setting. Hence, improving environmental hygiene and inadequate water sanitation, and health education for behavioral changes to personal hygiene would be crucial for effective control of the parasite infections.

**Electronic supplementary material:**

The online version of this article (10.1186/s13104-018-3166-3) contains supplementary material, which is available to authorized users.

## Introduction

Intestinal parasitic infection is one of the major childhood health problems in developing countries. According to the world health organization, over 270 million pre-school and over 600 million of school children live in areas where the parasites are intensively transmitted [1].

The disease is most prevalent among the lower social groups and in children whose parents are farmers that are likely to come in contact with the contaminated soil. This practice encourages the transmission of the parasites through penetration of the infective larvae present in the soil, and through direct or indirect fecal-oral transmission [2]. Despite there are availability of chemotherapy and control measures, intestinal nematode infections rank among the most wide spread of soil transmitted intestinal parasites [3]. Younger children are predisposed to heavy infections with intestinal parasites because of having not fully developed immune systems and also habitually they play in fecal contaminated soil [4].

Inadequate water sanitation and hygiene are responsible for a major proportion of the burden of disease and death. Intestinal parasitic agents increase in polluted environments such as refuse heaps, gutters and swage units in and around human dwelling and living conditions of the people in crowded or unhealthy situation [4, 5].

The symptoms of parasitic infection include anemia, asthma, diarrhea, digestive disorders, fatigue, low immune system, nervousness and skin rash. Apart from causing morbidity and mortality, infections with intestinal parasites have been associated with stunting, physical weakness, low education achievements, poor reproductive health, and low economic development [6, 7]. Furthermore, chronic intestinal parasitic infections have become the subject of speculation and investigation in relation to the spreading and severity of other infectious diseases such as human immunodeficiency virus (HIV) and leprosy [8].

In Ethiopia, intestinal parasites are widely distributed. Most of the intestinal parasites such as *A. lumbricoides* and *T. trichiura* showed wider distribution [9, 10]. A study done in North West Ethiopia showed higher prevalence of *H. nana* (13.8%), *E. histolytica/dispar* (9.2%) and *A. lumbricoides* (5.9%) [8]. The problem is more severe in the region as evidenced by a study done in Delgi Ethiopia that showed very high prevalence of *A. lumbricoides* (48%), *G. lamblia* (41.9%), *E. histolytica/dispar* (27.3%), *S. mansoni* (15.9%), and Hookworm (11.5%) [9]. In Jimma Ethiopia 26.3% of *S. mansoni* prevalence was documented [10]. Another study conducted in Eastern part of Ethiopia also found nine species of intestinal helminths with an overall prevalence of 27.2% [11]. However, there are still several localities for which epidemiological information is not available including the study area. Moreover, under-5 years of age children are more prone to intestinal parasites because of the low immunity they have in this stage that needs special care and follow up [12]. Therefore, the objective of this study was to assess the prevalence of intestinal parasitic infections among under-five children in Debre Birhan. This could help health planners to overcome the existing limitations and decrease the mortality in under-five children.

## Main text

### Methods

#### Study area, population and study subjects

A cross-sectional study design was conducted among under-five children in Debre Birhan town, February 2014. Debre Birhan is situated at an altitude of 2840 m above sea level with mean annual temperature that ranges from 10 to 16 °C. The population of the town is 81,775. All under-five children who came to the Debre Birhan referral hospital during the study period for medication were included because of more prone to intestinal parasites as a result low immunity they have in this stage that needs special care and follow up. Serious health problem and use of antihelminthic drugs within 1 month before screening were used as exclusion criteria from the study. However, there was no child excluded since we did not find children with these conditions. The sample size was determined using single population proportion formula based on the following assumptions: 17.3% prevalence of intestinal parasites among under 5 years children in Gondar, Ethiopia [13], 95% confidence interval and 5% marginal error, and by adding 10% compositions for the non-response rate. Hence, a total of 247 study participants were included in this study and selected consecutively based on the arrival of their hospital visit.

#### Data collection and laboratory diagnosis

Data of socio-demographic and possible risk factors were collected by two trained data collectors using structured questionnaire from the parents or guardians of the children. The questionnaire was developed by the authors of this study (Additional file 1). It was pretested at nearby health center (Debre Birhan Health Center) on 5% of samples calculated to include in this study (twelve under-five children) to improve the quality of data collection tool. After the pretest, the questionnaire was modified and corrected to make more appropriate tool for the data collection. Moreover, expert review was included in the development of the instrument to align with the theoretical framework guiding the study. After the pretest and expert inputs, the tool was modified for proper utilization.

Stool specimens, one for each study participant, were collected from the children using clean plastic containers. Microscopic examination was performed using Olympus microscope (CX21FS1, Olympus Corporation, Philippines) as soon as the specimen was collected. A combination of direct microscopy and the formol-ether concentration technique were used to detect the presence of ova, larvae, trophozoite, and cyst of parasites. Briefly, an estimated 1 g of faeces was emulsified using applicator stick in about 4 mL of 10% formol-saline contained in a screw-cap tube. A further 3–4 mL of 10% v/v formol-saline was added to the tube and mixed well and sieved. The sieved suspension was collected in a beaker. The suspension was transferred to a conical (centrifuge) tube. About 4 mL of diethyl ether was added to the conical tube, mixed for 1 min, and centrifuged at 3000 rpm for 1 min. After centrifuging, the tube was inverted to discard the ether, faecal debris, and formol water. The sediment was re-suspended and transferred to a slide, and covered with a cover glass. Finally, the preparation was examined microscopically using the 10× and/or 40× objectives [14]. Well experienced laboratory technologists who worked in Debre Birhan referral hospital did the stool specimen diagnosis. To ensure the quality of the investigation, the two readers read the slides independently and their readings were compared. Discordant were immediately resolved with discussion of each other and in consultation with the investigators.

#### Data analysis

Data were entered and analyzed using statistical package for social sciences (SPSS) version 20. Summary statistics (frequencies, proportions) in the form of tables, texts and figures were used to express the findings. The Pierson Chi square and related P values were used to check the presence of association between dependent and independent variables. P < 0.05 was considered as significant association.

#### Ethical clearance

This study was reviewed and approved by the Ethical Review Committee of Haramaya University. Official permission was obtained from Debre Birhan referral hospital. Written informed consent was obtained from the parents/guardians of the children. Those children who were positive for intestinal parasites were linked to the clinicians from Debre Birhan referral hospital to be treated. Data were confidential and used only for the purpose of this study.

### Results

#### Socio-demographic characteristics

A total of 247 children were selected for investigation. Of these, 129 (52.2%) were females and 101 (40.9%) were between age group 3–5 years. Children from mothers with illiterate, primary and, secondary and above education level were accounted 69 (27.9%), 96 (38.9%) and 82 (33.2%), respectively. One hundred two (41.3%) children were from families having monthly income between 1000 and 2000 Ethiopian Birr (Table 1).

**Table 1:** Socio-demographic characteristics of the under-five studied children living in Debre Birhan, Ethiopia, 2014

Characteristics	Category	Numbers	Percentage
Sex			
Female		129	52.2
Male		118	47.8
Age in year			
≤ 2		78	31.6
2–3		68	27.5
3–5		101	40.9
Monthly income of parents			
< 1000 Ethiopian Birr		65	26.3
1000–2000 Birr		102	41.3
> 2000 Birr		80	32.4
Maternal education			
Illiterate		69	27.9
Primary		96	38.9
Secondary and above		82	33.2

#### Parasitic infections and associated factors

**Fig. 1:**
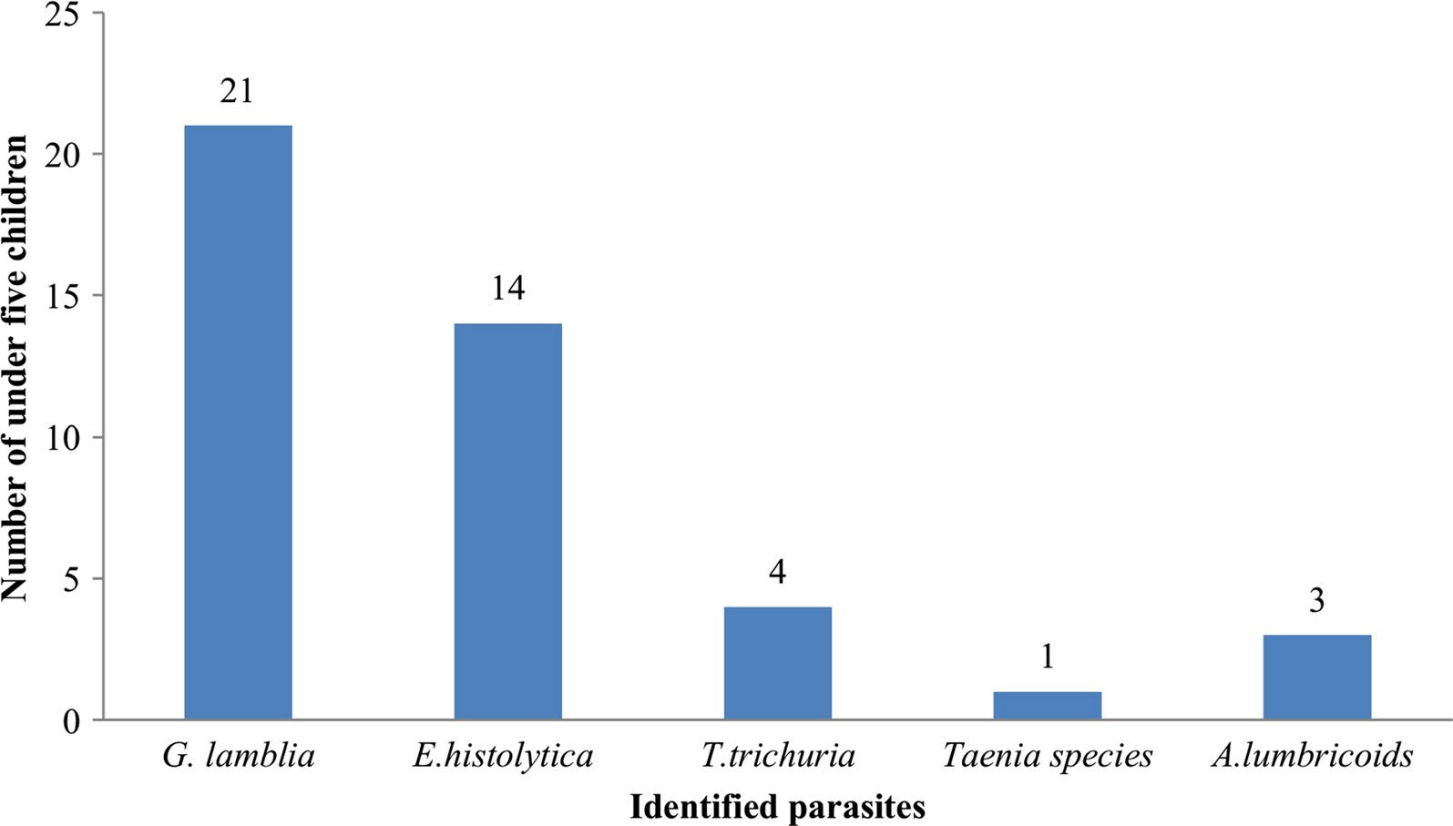
Identified intestinal parasites among under-five children, Debre Birhan, 2014

Among the 247 children participated in this study, 43 (17.4% [95% CI 12.7–22.1%]) children were infected with at least one or more parasites. Protozoa parasites, 35 (14.2% [95% CI 9.9–18.5%]), were the most dominant parasitic infections among the children. *G. lamblia* and *E. histolytica* were the pathogenic protozoan parasites found with a prevalence of 21 (8.5% [95% CI 5.0–8.0%]) and 14 (5.7% [95% CI 2.8–8.6%]), respectively. Eight (3.2% [95% CI 1.0–5.4%]) children were infected with helminth parasite(s). Double infection (*G. lamblia* and *E. histolytica*) was found in only five children. The distribution of identified parasites among under-five children is described in Fig. 1.

About 32% of the children with poor hand washing practice had significantly higher parasitic infections (X^2^: 17.5, 95% CI 26.2–37.8%, P < 0.001). Those who had no nail trimming (28.3%) were more infected (X^2^: 13.6, [95% CI 22.7–33.9%, P < 0.001). Similarly, 27.5% of the children born from illiterate mothers were highly infected (X^2^: 7.0, 95% CI 21.9–33.1%, P 0.03). Drinking water from river source was rated as the highest associated risk with 36.8% of parasitic infections (X^2^: 6.5, [95% CI 30.8–42.8%, P 0.039) (Table 2).

**Table 2:** Distribution of intestinal parasite among under five children in relation with socio-demographic and hygiene practices, Debre Birhan, Ethiopia, February, 2014

Variables	Category	Parasite infection		X^2^	P value
		Positive	Negative		
Sex					
Female		23	106	0.033	0.855
Male		20	98		
Frequent hand washing practice					
Yes		17	148	17.5	0.00
No		26	56		
Eating unwashed vegetable					
Yes		8	43	0.133	0.716
No		35	161		
Nail trimming					
Yes		15	133	13.6	0.00
No		28	71		
Maternal education					
Illiterate		19	50	7.0	0.03
Primary		14	82		
Secondary and above		10	72		
Source of drinking water					
Pipe		30	172	6.5	0.039
Well		6	20		
River		7	12		
Age in years					
≤ 2		14	64	0.042	0.979
2–3		12	56		
3–5		17	84		

*X*^*2*^ Chi square

### Discussion

Intestinal parasites are one of the leading causes of death among children in the developing countries. Hence, adequate information about the prevailing state is an important epidemiological tool in evaluating existing or new intervention programs [15]. In this study, the overall prevalence of intestinal parasitic infection was 17.3%. This is in line with findings from Gondar Ethiopia (17.3%) and from Tanzania (15.1%) [13, 16]. The finding is lower as compared to studies done in Wondo Genet (85.1%) [17], in Yergalem hospital (49.5%) [18], and in Kenya (25.6%) [19]. This variation could be due to different geographical distribution of the parasites, timeline and implementation of different prevention and control measures. However, the finding of our study is still higher according to the national safe environment strategy in the extension program in Ethiopia.

Pathogenic protozoa infections are known to cause diseases in children [4, 20]. The transmission of these parasites occurs by ingestion of cysts through the fecal–oral route, either directly, via person to person contact or indirectly, via contamination of surface water or food [21, 22]. In the present study, *G. lamblia* was the predominant protozoan parasite with a prevalence of 8.5%. This causes malaise, abdominal cramps, weakness, weight loss, distention, and flatulence. Children are more liable to massive infection with severe clinical manifestations [21]. The more chronic stage is associated with vitamin B12 mal-absorption, disaccharides deficiency and lactose intolerance [23]. This study also found that *E. histolytica* was the second prevalent (5.7%) protozoan parasite among the study participants. The organism may invade the liver, lung and brain where it produces abscesses that result in liver dysfunction, pneumonitis, and encephalitis [22]. The highest prevalence of protozoa parasites could be due to contaminated water as this study has shown that significantly higher parasitic infections (36.8%) were found among under-five children who drank water from river source.

Public health interventions such as the provision of clean water, community health education, observation of food hygiene, and maintenance of functioning sanitation systems are fundamental to long-term intestinal parasite control [24]. In this study, about 32% of the parasitic infection of the children was due to poor hand washing practice of mothers. This problem is also reported in University of Gondar community school [8]. Moreover, under-five children born from illiterate mothers were more infected (27.5%). Similarly, nail hygiene and educational level of mothers were closely associated with the prevalence of intestinal infections [5, 6]. All these evidences have shown that there should be effective implementation of intervention activities to control the spread of intestinal parasitic infections in the setting.

### Conclusions

This study revealed that intestinal parasite infections, especially protozoan parasites, were prevalent that affect the health of under-five children in the setting. Although these findings are limited to one hospital, it may represent the population of the area because of the wide range of health service provision in the hospital. Hence, improving environmental hygiene and inadequate water sanitation, and health education for behavioral changes to personal hygiene would be crucial for effective control of intestinal parasitic infections.

## Limitations

As limitation, only stool concentration method was used because of budget shortage that restricted us not to use more advanced methods like Kato-Katz, Harada Mori and Baermann techniques that may hide the true burden of intestinal parasitic infections. Children are only recruited within a single month, and there may be seasonal fluctuations of the prevalence of intestinal parasite in the study area.

## Additional file

**Additional file 1.** Questionnaire.

## Abbreviations

CI: confidence interval
HIV: human immunodeficiency virus
X^2^: Chi square
