# Supplementary material for: Prevalence of intestinal parasitic infections in children under the age of 5 years attending the Debre Birhan referral hospital, North Shoa, Ethiopia
Source: BMC Res Notes. 2018 Jan 22;11:58. doi: 10.1186/s13104-018-3166-3 (PMC5778703; doi:10.1186/s13104-018-3166-3)
Supplement: Supplementary file 1 — Additional file 1. Questionnaire. [file 13104_2018_3166_MOESM1_ESM.docx]

## Additional file 1

## ANNEX. Questionnaire

General instruction: this tool is used to collect information for identification of the burden of parasites in under-five years of age children in Debre Birhan. For each question, there are response column and is coded accordingly in front of it.

Code number: ________________________

Part I. Socio- demographic characteristics and prevention practices

| No. | Questions | Response options | Skip |
| --- | --- | --- | --- |
| 1 | Sex | 1. Male 2. Female |  |
| 2 | Age | ____ years |  |
| 3 | Monthly income of parents | 1. < 1000 Ethiopian Birr 2. 1000-2000 Birr 3. >2000 Birr |  |
| 4 | Maternal education | 1. Illiterate 2. Primary 3. Secondary and above |  |
| 5 | Frequent hand washing practice | 1. Yes 2. No |  |
| 6 | Eating unwashed vegetable | 1. Yes 2. No |  |
| 7 | Nail trimming | 1. Yes 2. No |  |
| 8 | Source of drinking water | 1. Pipe 2. River 3. Well |  |

Part II. Laboratory analysis worksheet

| SN | Specimen ID | Date specimen collected | Date specimen examined | Result of stool examination | Parasite stage | Lab personnel examined the specimen | Signature | Remark |
| --- | --- | --- | --- | --- | --- | --- | --- | --- |
|  |  |  |  |  |  |  |  |  |
|  |  |  |  |  |  |  |  |  |
|  |  |  |  |  |  |  |  |  |
|  |  |  |  |  |  |  |  |  |
|  |  |  |  |  |  |  |  |  |
|  |  |  |  |  |  |  |  |  |
|  |  |  |  |  |  |  |  |  |
|  |  |  |  |  |  |  |  |  |
|  |  |  |  |  |  |  |  |  |
|  |  |  |  |  |  |  |  |  |
|  |  |  |  |  |  |  |  |  |
|  |  |  |  |  |  |  |  |  |
|  |  |  |  |  |  |  |  |  |
